# Yeast Beta-Glucans Ingestion Does Not Influence Body Weight: A Systematic Review and Meta-Analysis of Pre-Clinical Studies

**DOI:** 10.3390/nu13124250

**Published:** 2021-11-26

**Authors:** Marcelo M. Canaan, Juliana C. Reis-Canaan, Márcio G. Zangerônimo, Eric F. Andrade, Thais M. S. V. Gonçalves, Michel C. A. Pereira, Renato R. Lima, Vanessa Pardi, Ramiro M. Murata, Luciano J. Pereira

**Affiliations:** 1 Health Sciences Department, Universidade Federal de Lavras (UFLA), Lavras BR-37200-000, Brazil; marcelo.canaan@ufla.br (M.M.C.); reisjuliana@yahoo.com.br (J.C.R.-C.); ericfrancelinoandrade@gmail.com (E.F.A.); 2 Veterinary Science Department, Universidade Federal de Lavras (UFLA), Lavras BR-37200-000, Brazil; zangeronimo@ufla.br; 3 Agrarian Sciences Institute, Universidade Federal dos Vales do Jequitinhonha e Mucuri (UFVJM), Unaí BR-38610-000, Brazil; 4 Dentistry Department, Universidade Federal de Santa Catarina (UFSC), Florianópolis BR-88040-900, Brazil; thais.goncalves@ufsc.br; 5 Department of Nutrition, Universidade Federal de Lavras (UFLA), Lavras BR-37200-000, Brazil; deangelis@ufla.br; 6 Department of Statistics, Universidade Federal de Lavras (UFLA), Lavras BR-37200-000, Brazil; rrlima@ufla.br; 7 Department of Foundational Sciences, School of Dental Medicine, East Carolina University (ECU), Greenville, NC 27834, USA; pardiv19@ecu.edu

**Keywords:** fungi, yeast, beta-glucans, body weight, rodents, obesity

## Abstract

Dietary fiber supplementation has been studied as a promising strategy in the treatment of obesity and its comorbidities. A systematic review and meta-analysis were performed to verify whether the consumption of yeast beta-glucan (BG) favors weight loss in obese and non-obese rodents. The PICO strategy was employed, investigating rodents (Population), subjected to the oral administration of yeast BG (Intervention) compared to animals receiving placebo (Comparison), evaluating body weight changes (Outcome), and based on preclinical studies (Study design). Two reviewers searched six databases and the grey literature. We followed the PRISMA 2020 guidelines, and the protocol was registered on PROSPERO (CRD42021267788). The search returned 2467 articles. Thirty articles were selected for full-text evaluation, and seven studies remained based on the eligibility criteria. The effects of BG intake on body weight were analyzed based on obese (*n* = 4 studies) and non-obese animals (*n* = 4 studies). Even though most studies on obese rodents (75%) indicated a reduction in body weight (qualitative analysis), the meta-analysis showed this was not significant (mean difference −1.35 g—95% CI −5.14:2.45). No effects were also observed for non-obese animals. We concluded that the ingestion of yeast BG barely affects the body weight of obese and non-obese animals.

## 1. Introduction

Obesity is a complex disease in which genetic, immunometabolic and environmental factors are involved [1]. It is associated with considerable public health consequences [2], causing approximately 3.4 million deaths worldwide every year [3]. Hypertension, dyslipidemia, cardiovascular disease, cancer [4] and mainly diabetes mellitus are closely associated with obesity [5] and, due to its increasing prevalence and morbidity risks, this disease is considered a global health pandemic [3].

The importance of weight balance for health is emphasized by most medical societies [6,7]. It generally involves lifestyle changes with caloric restrictions and physical exercise [7,8]. There are relatively few drugs approved for obesity treatment, and some of them have important side effects that contraindicate their use or are financially unfeasible for the majority of the population [7]. Furthermore, it is known that losing weight and maintaining this loss is not always an easy task, with most individuals experiencing weight regains [8].

Along with lifestyle changes, soluble fiber ingestion seems to be a relatively inexpensive and applicable approach to improve blood glucose and body weight control in patients with overweight and obesity [9]. In this context, beta-glucans (BGs) are a diverse group of natural polysaccharides. Their effects vary according to molecular weight, tertiary structure, solubility and type of linkage branching [10]. BG chemical structures have a central linear β (1 → 3) ligation composed of D-glucose monomers linked by a β-glycosidic bond, with the ramification (1 → 4) found in those extracted from bacteria and plants, whereas β (1 → 6) are found in BGs extracted from fungi [11,12]. These fibers have a beneficial role in metabolic disorders due to their ability to form a viscous solution (decreasing carbohydrate and lipid absorption) and to ferment (acetate, propionate and butyrate) in the gut, influencing the intestinal mucosal immunity, in addition to the barrier integrity and function [10].

Traditionally, cereal BGs are known to cause metabolic benefits [13,14], whereas those from microorganisms improve immune responses [15,16]. However, recent studies from our group have demonstrated metabolic benefits after yeast BG (*Saccharomyces cerevisiae*) ingestion [17,18,19], in addition to the immunological enhancement [20,21]. However, the effects of fungal BG on body weight control are still unknown, and potentially controversial [22,23].

Before indicating alternative treatments for human patients, effectiveness criteria must be raised. The best evidence for clinical decision might be generated using systematic reviews of randomized clinical trials. However, animal studies evaluate more homogenous samples, with standardized feeding and environments. These models provide well-controlled in vivo results based on physiologically and genetic similar organisms, which increase our understanding of human diseases and isolated treatment effects [24]. In this sense, animal models are essential for establishing the applicability and safety of novel therapeutics prior to human consumption [25]. Consequently, before considering yeast BG as a potential adjuvant agent for treating obesity, it is important to verify whether there is sufficient evidence of its effects in pre-clinical studies. Thus, we hypothesized that BG intake would reduce body mass in an obesity model. Therefore, the objective of this systematic review was to evaluate the effect of yeast BGs on the body weight control of obese and non-obese rodents against placebo.

## 2. Materials and Methods

### 2.1. Registration Protocol and Study Design

This systematic review was registered on PROSPERO under registration number CRD42021267788. Besides, the present manuscript was organized based on PRISMA 2020 guidelines (Preferred Reporting Items for Systematic Reviews and Meta-analyses) [26].

### 2.2. Focused Question

The Participants (P), Interventions (I), Control (C) and Outcomes (O) (PICO) format was used to formulate the focused question: “Can the consumption of yeast BG (I) favor obese and non-obese rodents’ (P) weight loss (O) in comparison to placebo (C)?”.

### 2.3. Eligibility Criteria

#### 2.3.1. Search Strategy

We searched six different electronic databases (Embase, PubMed, Scielo, Science Direct, Scopus and Web of Science). The search was conducted until July 2021, using the following keyword combination: “yeast” and “beta-glucan” and “body weight”. Appropriate MeSH and entry terms (*Saccharomyces cerevisiae* or baker or sizofiran or lentinan or zimosan/and beta-glucan or β-glucan or beta glucans/and overweight or obesity or body mass index or weight loss or weight gain) were used. A similar strategy was employed in all databases. We also used keywords translated into Portuguese and Spanish in Scielo database. Furthermore, grey literature (Google Scholar, Proquest Dissertations and Thesis and Open Gray databases) and manual searches on the references of the included studies were also consulted. In all cases, no restrictions on the language or publication date were applied. Details of searches in each database are presented in Supplementary File S1. In some databases, the number of keywords was adapted to provide a broader search according to the available tools.

#### 2.3.2. Inclusion Criteria and Study Selection

For the present review, we selected only in vivo pre-clinical studies involving rodents (mice or rats), investigating the effects of oral yeast BG supplementation on body weight changes. Literature reviews, letters to Editors, reference guides, and studies conducted on other species of animal models besides rodents or using other sources of BG were excluded.

To avoid confounding factors, we only included studies investigating the isolated effect of yeast BG. In this sense, studies involving other fungal species (such as mushrooms), mixtures of the yeast BG into other foods, probiotics, short exposition (1 week or less), experiments with other disease models than rodent obesity (e.g., streptozotocin-induced diabetes before the final body weight evaluation, inflammatory and infectious diseases, cancer and irradiation) were also excluded. No restrictions were made regarding the sex, number of animals, purity, or dosage of yeast BG.

#### 2.3.3. Articles Selection and Data Extraction

Two researchers (M.M.C and J.C.R.C.) separately conducted the database searches and independently reviewed all titles and abstracts using Rayyan software (https://www.rayyan.ai/, accessed on 16 November 2021) and the Mendeley^®^ reference manager (www.mendeley.com, accessed on 16 November 2021). Studies that did not meet the eligibility criteria were excluded. Then, from the selected abstracts, the same investigators evaluated the full manuscripts based on these same criteria. The senior author (L.J.P.) made the final judgment when a consensus could not be reached by the two reviewers. Articles excluded after this phase with respective reasons can be seen in Supplementary File S2.

The data were independently collected by the same reviewers and the information was then cross-checked. Information including the authors, year of publication, study design, experimental period, source of BG, yeast species, animal characteristics (mice or rats), sex, initial and final body weight, statistical analysis, and main outcomes were assessed. When the weight values were graphed, we tried to contact the authors by email and, in cases of no feedback, we estimated the values using the Image J program (http://imagej.nih.gov/ij/, accessed on 16 November 2021).

#### 2.3.4. Risk of Bias (RoB) Assessment

We used the Systematic Review Centre for Laboratory Animal Experimentation (SYRCLE) RoB tool to evaluate the risk of bias. This document contain 10 entries, related to 6 types of bias: selection, performance, detection, attrition, reporting and other biases [27].

#### 2.3.5. Methodological Quality Assessment

We qualitatively analyzed the selected studies according to the Animal Research Reporting In Vivo Experiment (ARRIVE) guidelines [28]. This checklist contains a predefined grading for 20 categories [28,29]. The categories were represented by letters from “A” to “T”, with the domains A, D, K and N worth one point, and the remaining domains worth two points. The sum score varied from 0 to 36 points, and the maximum score for each category was calculated. We also calculated the Quality Score/Maximum Score ratio, defining three possible range coefficients in which scores below 0.5 were considered “poor”, from 0.5 to 0.8 “average”, and from 0.81 to 1 “excellent” [30].

### 2.4. Data Analysis

We assessed clinical and methodological heterogeneity by analyzing study characteristics such as species (rat or mouse), sex, dose of BG, time of use, outcome measurements or comparators to determine the possible body weight changes. We also separately evaluated the animals’ BG consumption into two groups (not obese and obese animals), with their respective sex and weight-matched controls.

Meta-analysis was performed using the META package [31] of R statistical software [32]. A random effects model was used for the meta-analysis. The summary of the effect measure was depicted in a forest plot, containing the mean difference (MD) and 95% confidence intervals (CIs). For each study, the mean value, standard deviation and sample size were reported for both experimental and control groups, separating for obese and non-obese subgroups. The publication bias was quantitatively evaluated by funnel plots to identify and to avoid asymmetries in the selected studies [33].

## 3. Results

### 3.1. Study Selection and Characteristics

**Figure 1:**
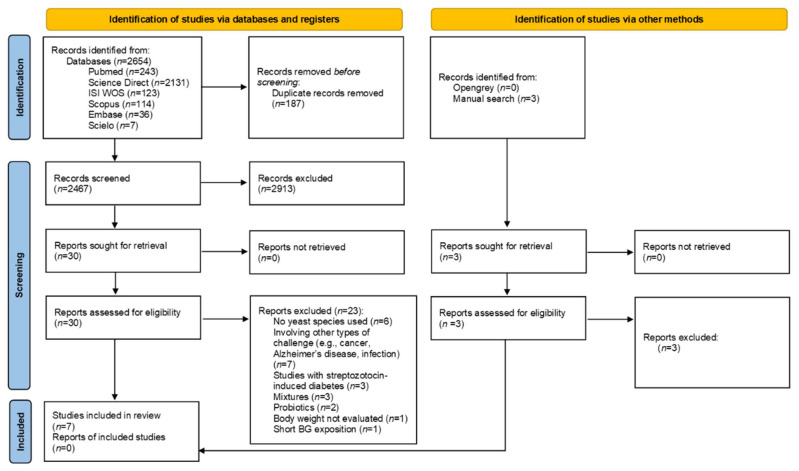
Flow diagram of the screened articles adapted from the PRISMA statement.

The flowchart diagram of this review is outlined in Figure 1. A total of 2654 reports was initially identified after searching in all databases. After removing duplicates, 2467 studies had their titles and abstracts read and 30 potential references were appraised. Of these 30 articles, 23 were excluded because: not used yeast species (*n* = 6); involving other types of challenge (e.g., cancer, Alzheimer’s disease, infection) (*n* = 7); used model streptozotocin-induced diabetes (*n* = 3); administered BG mixed with other agents (*n* = 3); administered probiotics (*n* = 2); not evaluated body weight (*n* = 1); and performed short BG exposition (*n* = 1). Three additional articles were found by manually searching, but they were excluded after full-text reading (Supplementary File S2). Finally, seven studies were selected. Three of the studies only investigated non-obese animals [34,35,36], whereas another three evaluated only obese animals [37,38,39]. One article evaluated both normal-weight and obese animals [19]. Thus, each analysis contained four studies. Seven selected articles generated eight entries.

### 3.2. Results for Individual Studies

The characteristics of the seven studies included in the present study are described in Table 1. In all cases, yeast was the only source of BG, and body weight was a secondary outcome. The only yeast used in all studies was *Saccharomyces cerevisiae*, and in only one of them, the purity found was less than 50% [39].

The majority of samples (62.5%) involved rats [19,34,35,36]. In studies involving non-obese animals, only rats were used, whereas in 37.5% of the studies involving obese rodents, mice were used [37,38,39]. Half of the studies only evaluated males [19,35,37], whereas 37.5% analyzed both males and females [34,36,38], and one study was performed only using females [39]. In none of the studies was a metabolic cage used.

In non-obese animals, the intervention period ranged from 14 to 91 days, and the dose comprised values between 2 and 2000 mg/kg body weight/day, [19,34,35,36]. In obese animals, the intervention period ranged from 28 to 49 days, and the doses varied from 25 to 450 mg/kg [19,37,38,39] applied three times a week (mean of 193 mg/kg body weight/day), as described by Shituleni et al. [39].

For non-obese animals, none of the studies showed statistically significant weight loss. Nonetheless, for obese animals, statistically significant differences were reported in 75% of the studies [37,38,39].

### 3.3. Bias of Risk and Methodological Quality Assessments

In the risk of bias analysis, no concerns were found for “sequence generation” and “random outcome assessment”, because all studies (100%) provided a proper description and objective body weight analyses were carried out. A low risk of bias was found for most studies in terms of “baseline characteristics” (87.5%), “selective outcome reporting” (87.5%), and “incomplete outcome data” (75%) domains. However, considering “other bias” and “allocation concealment”, only some of the studies succeeded, with 50% and 37.5%, respectively. None of the studies provided clear information about “random housing”, “blinding of participants and personnel” or “blinding of outcome assessment” (Table 2).

The total score obtained through ARRIVE guidelines ranged from 28 to 30 points (mean score 29.37 ± 1.89) (Table 3) within a maximum score of 36. Of the 20 entries evaluated, 14 categories (70%) (A, C, D, E, G, H, K, L, M, N, O, P, S and T) scored as “excellent” (between 0.8 and 1.0), whereas 6 (30%) (B, F, I, J, Q and R) were classified as “average” (between 0.5 and 0.8). No category was classified as “poor” (below 0.5) (Table 3).

### 3.4. Meta-Analysis Results

**Figure 2:**
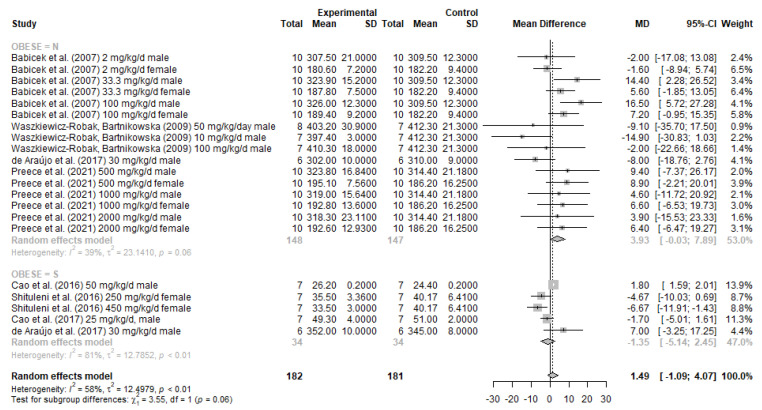
Forest plot and meta-analysis of BG on the body weight of obese and non-obese rodents [19,34,35,36,37,38,39].

In general, heterogeneity among studies was significant (I^2^ = 58%; *p* < 0.01), and for this reason, random effects models were preferred (Figure 2). Non-significant mean differences were found between BG and controls for both non-obese (MD: 3.93 g, τ^2^ = 23.14; 95% CI: −0.03 to 7.89) and obese animals (MD: −1.35 (τ^2^ = 12.7852; 95% CI: −5.14 to 2.45).

## 4. Discussion

The findings of the present study indicate that the oral consumption of yeast BG is not associated with a significant reduction in body weight for either non-obese or obese animals, rejecting our initial hypothesis. Although 75% of primary studies evaluating obese animals indicated significant body weight reduction, after meta-analysis, this result was not significant (adjusting for samples sizes and respective study relative weight in the model). BG did not influence body weight either both qualitative or quantitative analyses.

In the present study, genetic background, obesity induction models, time after induction of obesity to start BG ingestion, and duration of BG treatment varied widely among the studies, which made translational comparisons difficult [25]. In addition, the experimental findings suggest that the effects of BG vary according to the route of administration, average molecular weight, and differences in dose, purity and water-solubility [12,40]. Recently, Markovina et al. summarized the results and efficacy of different sources of BG and highlighted that, in general, it is complicated to provide recommendations because clinical details of BG type and dosage are not always clear [41]. Due to these considerations, constant improvements are needed to adjust preclinical models, so that they can significantly reflect clinical observations and processes [25].

BGs reduce glucose and lipid absorption by forming a gelatinous barrier in the intestine [19,42] whereas, a consequent reduction in body weight was expected. Although the barrier effect inherent to fibers has been described and valued, it can be assumed that it is transient [43]. In this sense, the metabolic benefits of BG ingestion in relation to blood glucose and lipoprotein profile might be related to other pathways in addition to the barrier effect. Fiber-rich diets increase the intestinal production of short-chain fatty acids (SCFA) such as acetate, propionate and butyrate, leading to higher microbial diversity in addition to reductions in inflammation and insulin resistance [44,45]. Although many studies have sought an association between prebiotics/probiotics and body weight reduction, this outcome is generally quite discrete, even in long-term supplementation [46]. Therefore, the use of such agents should be considered not for body weight reduction, but to preventively control insulin resistance and dyslipidemia. In the context of metabolic disorders, these effects are of clinical relevance [46]. However, the mechanisms by which BGs beneficially regulate the intestinal microbial population remain under debate [47].

In models with obese animals, we found that yeast BG consumption reduced body weight in 75% of primary studies (based on qualitative analysis). However, it is important to consider that even though qualitative analysis indicated a significant reduction in body weight for obese animals, the amount of weight loss seems quite small and possibly irrelevant. It is known that BG may induce satiety, contributing to explaining the slight reduction in body weight observed after BG ingestion. Shituleni et al. observed that obese mice supplemented with yeast BG had lower food intake and significant weight loss compared to the placebo group [39]. Some properties of BG, including gut swelling, increased chyme viscosity and consequent delay in gastric emptying, could contribute to food intake reductions [48,49]. Thus, the presence of fibers in the stomach can generate an early feeling of fullness, although it is short in duration [43].

Another important factor may be attributed to changes in the gut microbiota after BG consumption [38,50]. Treatment with yeast BG suppresses gut inflammation, altering the microbiota composition and ultimately increasing the immune-regulatory SCFA production [51]. Coexisting microorganisms in the digestive tract can modify various chemicals, triggering host reactions that modulate important effects on immunity and metabolism [52]. The gut microbiota can be modulated by the diet and lead to changes in the balance between different bacterial phyla. Thus, those with greater capacity to extract energy from certain macronutrients such as fiber predominate [53]. SCFA and polysaccharide metabolites, obtained through the enzymatic action of the intestinal microbiota, play important roles in gene expression, proliferation, chemotaxis, differentiation and apoptosis of animal cells [54]. Changes in energy extraction capacity, even if slight, can prove to be significant over time, impacting on body weight [53]. More possible evidence of BG effects on the microbiota is the increased abundance of *Akkermansia muciniphila*, a bacterium inversely associated with insulin resistance [38].

Despite being more studied in research involving immunological parameters, there is parallel evidence that yeast BG performs similar metabolic activity to cereal-BG such as oats and barley [18,55]. It is also described that BGs cause a delay in the digestive enzyme action on starch. This could result in a reduction in carbohydrate absorption, and, consequently, a reduction in blood glucose [55]. β-glucans extracted from yeast are mostly insoluble glucose monomers, presenting β-1, 3 d-glucose linkages and β1, 6 side branches [56,57]. Solubility can be induced by acid degradation method [58]. The main component of β-glucan from the yeast cell wall is a slightly branched, high-molecular (1 → 3)-β-D-glucan, with about 3% of β (1 → 6) branching [56]. Yeast β-glucans enhance bowel motility and intestinal obstipation [58,59,60]. The main action mechanism after ingestion involves contact with pattern recognition receptors (PPRs) in the intestinal cells, which internalize fragments that will interact and activate the immune system [61]. Yeast BG also interferes with liver lipid metabolism, expressively changing the transcriptional profiles, and leading to a reduced lipid accumulation in obese mice livers [37]. Additionally, this fiber can modulate the immune response, decreasing the insulin resistance linked to obesity [62]. Taken together, these facts can ultimately contribute to weight loss. Thus, there are many different and indirect ways in which BG can affect the metabolism and, notably, the body weight.

Currently, type 2 diabetes and obesity are considered subclinical inflammatory states, and BG administration is among the strategies with the power to mitigate inflammatory conditions, by reducing the production of pro-inflammatory cytokines. Such results may lead to an improvement in the metabolic status [38]. However, it is necessary to be aware that BG structural differences depend on the source, and can promote changes in their properties, resulting in specific different outcomes [12]. In this systematic review, a statistically significant difference in body weight was not found in any of the studies involving both obese and non-obese animals.

Regarding the risk of bias assessed by the SYRCLE RoB tool [27], our results are in agreement with those found by other systematic reviews of preclinical studies [63,64]. In studies involving animals, it is relatively common to observe biases due to the lack of blinding and randomization [64], essential items to avoid subjective outcome measurements and to reduce measurement bias [65]. Related to the quality criteria assessed through the ARRIVE guidelines [28], we observed that the categories “study design” and “sample size” received the lowest ratings. Evaluating the quality of reporting interventional animal studies, Ting et al. [66] reported that none of their 41 selected studies described sample size calculations, as well as the reporting of randomization and assessor blinding which occurred in 17.1% and 29.3% of the studies, respectively. Although sample calculations were not demonstrated in any article, it is assumed that most studies performed it, because for approval from animal research ethics committees, this definition is usually mandatory in order to avoid animal misuse. However, due to their importance, these details should not be omitted [66]. In this sense, limitations of the present research protocol relate to a lack of information in several studies, such as sample size calculation, randomization, and lack of blinding. However, overall scores of the ARRIVE guidelines indicated an average of 82% (Table 3).

BG ingestion seems to be harmless [34]. We did not find toxicity or side effect reports in any study. Even in humans, high doses such as 15 g/day have already been used without damage, which proves the safety of this supplement [67]. Flatulence, diarrhea and abdominal discomfort have been reported for humans, but they are usually discreet, and it is not necessary to suspend the treatment [67,68].

Systematic reviews can facilitate the translation of research results from animals to humans [69]. Animal studies, however, differ from clinical studies in some aspects, such as the diversity of animal species, experimental design and study characteristics. According to the Collaborative Approach to Meta-Analysis and Review of Animal Data from Experimental Studies, CAMARADES (www.camarades.info, accessed on 16 November 2021), heterogeneity over 60% is very common in meta-analyses using animal studies. Instead of abandoning meta-analyses, random effects models are suggested, which better fit the variation in animal studies [70]. Additionally, these animal studies generally present a reduced sample (around 10 animals per group), and slightly different studies of an individual intervention are often performed across many laboratories. There is also a great emphasis on minimizing variance through the use of inbred strains, pathogen-free environments, and specific handling conditions. Thus, it is recommended to random effects models when I^2^ values are greater than 50% [71].

## 5. Conclusions

Yeast beta-glucans ingestion does not significantly influence the body weight of obese and non-obese rodents.

## Figures and Tables

**Table 1:** Data extraction of the selected non-obese and obese animal studies.

References	Animal Model (Specie, Sex, Age) and Randomization	Specie and Purity	Groups and Dose of BG	Experimental Period	Body Weight Evaluation	Statistical Analysis ^#^	Effects of BG on the Body Weight	Obesity Status
Babíček et al. (2007) [34]	Acute model: Brl- Han:WIST@Jcl rats male and female 5 weeks old Sub-chronic model: SPF Fisher CDF (F-344)/CrlBR rats (sub-chronic model) male and female 5–6 weeks old	*Saccharomyces cerevisiae* Purity: >75%	Acute toxicity study: Control group Intervention group: dose: 2000 mg/kg body weight (BW)/day *n* = 10 (5/group) Sub-chronic toxicity study: Control group Intervention groups: dose: 2 mg/kg BW/day dose: 33.3 mg/kg BW/day dose: 100 mg/kg BW/day *n* = 120 60 male and 60 female were randomly selected according to weight criteria and allocated in 4 groups (ou 10/sex/group ?)	14 days 91 days	once a week	*t*-test ANOVA	no statistically significant difference	non-obese
Waszkiewicz-Robak et al. (2009) [35]	Wistar rats male age not mentioned	*Saccharomyces* *cerevisiae* Purity: 92%	Control: standard diet Intervention groups: BG 10 mg/kg BW/day BG 100 mg/kg BW/day Dried spent brewer’s yeast 50 mg/kg BW/day *n* = 29 (dried spent brewer’s yeast group = 8; the other = 7/group). After eating, all rats were fed ad libitum diet containing cholesterol	42 days	daily	ANOVA	no statistically significant difference	non-obese
Araújo et al. (2017) [19]	Wistar rats male 3 weeks old	*Saccharomyces cerevisiae* Purity: >60%	Group C: control diet Group CB: control diet treated with BG 30 mg/kg/day Group O: obese, high-fat diet Group OB: obese, high-fat diet treated with BG 30 mg/kg/day *n* = 24 (6/group)	28 days (after 60 days of obesity induction)	after 60 days of obesity induction and after 28 days of intervention	paired *t*-test	no statistically significant difference Obs: comparison of Groups CB × C	non-obese
Preece et al. (2021) [36]	Han:WIST rats male and female age not mentioned	*Saccharomyces cerevisiae* 90%	40 male and 40 female divided separately into 4 groups: 0 (control group) BG 500 mg/kg BW/day BG 1000 mg/kg BW/day BG 2000 mg/kg BW/day *n* = 80 (10/sex/group)	28 days	twice a week	one-way ANOVA followed by Duncan’s multiple range test	no statistically significant difference Obs: transitorily between 21 and 24 days it was a difference in weight gain in female using middle-dose (1000 mg)	non-obese
Cao et al. (2016) [38]	C57BL/6 mice male and female 7 weeks old	Baker’s yeast β-(1 → 3)-glucan (BYG) (*Saccharomyces cerevisiae*) Purity: 99%	ND group (normal diet), *n* = 10 HF group (high-fat), *n* = 30 PRE group (high-fat + BG 50 mg/kg/day), *n* = 10 After 30 days, streptozotocin-induced diabetes in mice of the HF and PRE groups. Then, HF group was subdivided into three new groups. MODEL group (high-fat diet + saline), *n* = 8 POST group (high-fat diet + BG 50 mg/kg/day), *n* = 8 MET (high-fat diet + metformin 50 mg/kg/day), *n* = 8	first phase: 30 days (period of evaluation) streptozotocin diabetes induction: from day 31 to day 40 second phase: day 41 to day 120	at the beginning and end of the first phase (30 days)	Paired-samples *t*-test (among two groups) and one-way ANOVA with Bonferroni’s post hoc test (among multiple groups)	statistically significant decrease Obs: body weight of the PRE (high-fat/BG) group was significantly lowered compared with HF group (high-fat) in the day 30 (first phase), before streptozotocin-induced diabetes.	obese
Shituleni et al. (2016) [39]	ICR mice female 4 weeks old	*Saccharomyces cerevisiae* Purity: >25%	Group A: control diet Group B: high-fat diet (HFD) Group C: HFD + 250 mg/kg yeast polysaccharide (YPS) 3 times a week HFD + 450 mg/kg YPS 3 times a week *n* = 60 (15/group) (*n* = 7/group for the body weight evaluation)	49 days	once a week	one-way ANOVA followed by the Student–Newman–Keuls post hoc test	statistically significant decrease	obese
Cao et al. (2017) [37]	ob/ob mice C57BLKS.B6.V-Lepob/Nju male 11–12 weeks old	Baker’s yeast β-(1 → 3)-glucan (BYG) (*Saccharomyces cerevisiae*) Purity: 99%	Control group: water Treated group: BYG 25 mg/kg/day *n* = 14 (7/group)	28 to 35 days with BYG diet; sacrificed at the age of 4−5 months	at the beginning and after 25 days of use of the BYG	Student’s *t*-test	statistically significant decrease	obese
Araújo et al. (2017) [19]	Wistar rats male 3 weeks old	*Saccharomyces cerevisiae* Purity: >60%	Group C: control diet Group CB: control diet treated with BG 30 mg/kg/day Group O: obese, high-fat diet Group OB: obese, high-fat diet treated with BG 30 mg/kg/day *n* = 24 (6/group)	28 days (after 60 days of obesity induction)	after 60 days to obesity induction and after 4 weeks of intervention	paired *t*-test	no statistically significant decrease Obs: comparison of Groups OB × O	obese

Statistical Analysis^#^: Identification of the test used by the authors. ANOVA—analysis of variance. BG—beta-glucan. BW—body weight. BYG—baker’s yeast β-(1 → 3)-glucan. HFD—high-fat-diet. MET—metformin. OB—obese. SPF—specific-pathogen-free. YPS—yeast polysaccharide. Fisher (CDF) — https://www.criver.com/products-services/find-model/fischer-cdf-rat?region=3621 (accessed on 16 November 2021).

**Table 2:** Assessment of the risk of bias in included studies.

Studies	A	B	C	D	E	F	G	H	I	J
Non-obese animals										
Babíček et al. (2007) [34]	+	+	-	-	-	+	-	+	+	?
Waszkiewicz-Robak et al. (2009) [35]	+	+	-	-	-	+	-	+	+	?
Araújo et al. (2017) [19]	+	+	+	-	-	+	-	+	+	+
Preece et al. (2021) [36]	+	+	-	-	-	+	-	+	+	+
Obese animals										
Cao et al. (2016) [38]	+	-	-	-	-	+	-	?	+	+
Shituleni et al. (2016) [39]	+	+	+	-	-	+	-	-	?	?
Cao et al. (2017) [37]	+	+	-	-	-	+	-	+	+	?
Araújo et al. (2017) [19]	+	+	+	-	-	+	-	+	+	+

A: Sequence generation. B: Baseline characteristics. C: Allocation concealment. D: Random housing. E: Blinding of participants and personnel. F: Random outcome assessment. G: Blinding of outcome assessment. H: Incomplete outcome data. I: Selective outcome reporting. J: Other bias. +: Yes (Low risk of bias). Unclear. -: No (High risk of bias).

**Table 3:** Scores of quality assessment according ARRIVE guidelines of the animal models in included studies.

Studies		ARRIVE Items																			
	A	B	C	D	E	F	G	H	I	J	K	L	M	N	O	P	Q	R	S	T	Total
Non-Obese Animals																					
Babíček et al. (2007) [34]	1	1	2	1	2	1	1	2	2	1	1	2	1	1	2	2	2	2	2	1	30
Waszkiewicz-Robak et al. (2009) [35]	1	1	2	1	2	1	2	1	1	1	1	2	2	1	2	2	1	1	2	1	28
Araújo et al. (2017) [19]	1	1	2	1	2	1	2	2	2	1	1	1	2	1	2	2	1	2	1	2	30
Preece et al. (2021) [36]	1	1	2	1	1	1	1	1	2	1	1	2	2	1	2	2	2	2	2	2	30
Obese Animals																					
Cao et al. (2016) [38]	1	2	2	1	2	1	2	2	0	1	1	2	2	1	1	2	1	1	2	2	29
Shituleni et al. (2016) [39]	1	1	2	1	2	1	2	2	2	1	1	2	2	1	1	2	1	1	2	2	30
Cao et al. (2017) [37]	1	1	2	1	2	1	2	2	0	1	1	2	2	1	1	2	1	1	2	2	28
Araújo et al. (2017) [19]	1	1	2	1	2	1	2	2	2	1	1	1	2	1	2	2	1	2	1	2	30
Category Score (Quality Obtained)	8	9	16	8	15	8	14	14	11	8	8	14	15	8	13	16	10	12	14	14	235
Maximum Score Expected (Quality Expected)	8	16	16	8	16	16	16	16	16	16	8	16	16	8	16	16	16	16	16	16	288
Ratio Quality Score/Maximum Score	1	0.56	1	1	0.94	0.50	0.88	0.88	0.69	0.50	1	0.88	0.94	1	0.94	1	0.63	0.75	0.88	0.88	0.82

A: Title. B: Abstract. C: Introduction—background. D: Introduction—objectives. E: Methods—ethical statement. F: Study design. G: Experimental procedure. H: Experimental animals. I: Housing and husbandry. J: Sample size. K: Allocation. L: Experimental outcomes. M: Statistics. N: Results—baseline data. O: Number analyzed. P: Outcomes and estimations. Q: Adverse events. R: Discussion—interpretation/scientific implications. S: General applicability/relevance. T: Funding. Total: Total score obtained by each manuscript out of a maximum of 36 points.

## Data Availability

Data are available upon request.

## Supplementary Materials

The following are available online at https://www.mdpi.com/article/10.3390/nu13124250/s1, Supplementary File S1: Search strategy on databases. Supplementary File S2: Articles excluded and reasons for exclusion (databases *n* = 20; manual search *n* = 3).
