# Supplementary material for: Yeast Beta-Glucans Ingestion Does Not Influence Body Weight: A Systematic Review and Meta-Analysis of Pre-Clinical Studies
Source: Nutrients. 2021 Nov 26;13(12):4250. doi: 10.3390/nu13124250 (PMC8707765; doi:10.3390/nu13124250)
Supplement: Supplementary file 1 [file nutrients-13-04250-s001.zip › nutrients-1463624-supplementary.pdf]

Supplementary File S1. Search strategy on databases

**PUBMED**<br><br>(“yeast” OR “*saccharomyces*” OR “baker” OR “zymosan” OR “Sizofiran” OR “Lentinan”) AND (“beta-glucan” OR “beta glucans” OR “β-glucan”) AND (“body weight” OR “obesity” OR “overweight” OR “weight loss” OR “weight gain” OR “BMI” OR “body mass index”)<br>Use of MeSH/entry therms
**SCOPUS**<br><br>TITLE-ABS-KEY (“yeast” OR “*saccharomyces*” OR “baker” OR “zymosan” OR “sizofiran” OR “lentinan”) AND TITLE-ABS-KEY ("beta-glucan" OR "beta glucans" OR "β-glucan") AND TITLE-ABS-KEY ("body weight" OR "obesity" OR "overweight" OR "weight loss" OR "weight gain" OR "BMI" OR "body mass index")
**WEB OF SCIENCE**<br>**COCHRANE**<br>**PROQUEST**<br><br>TOPIC: (“yeast” OR “*Saccharomyces*” OR “Zymosan”) AND TOPIC: (“beta-glucan” OR “beta glucans”) OR “βglucan”) AND TOPIC: (“body weight” OR “BMI”) OR “body mass index”)
**EMBASE**<br><br>“Yeast” AND “beta glucan” AND “body weight”
**ScienceDirect**<br><br>(“yeast” OR “*Saccharomyces*”) AND (“beta glucan” OR “β glucan”) AND (“body weight” OR “BMI” OR “body mass index” OR “overweight” OR “obesity”)
**Scielo**<br><br>(“β-glucans”) AND (“weight”)
**Opengrey**<br><br>(“yeast” AND “beta glucan” AND “body weight”)

Supplementary File S2. Articles excluded and reasons for exclusion (databases n = 20; manual search n = 3)

Author	Reason
Kuroiwa et al., (2005) [1]<br>Kimura et al., (2007) [2]<br>Chen et al., (2011) [3]<br>Ubaidillah et al., (2015) [4]<br>Chen et al., (2018) [5]<br>Silva et al., (2018) [6]	Non-yeast species used
Shin et al., (2007) [7]<br>Miranda et al., (2008) [8]<br>Shi et al., (2016) [9]<br>Shao et al., (2019) [10]<br>Gudi et al., (2020) [11]<br>Hiramoto et al., (2020) [12]<br>Xu et al., (2020) [13]	Studies involving other types of challenge<br>(e.g. cancer, Alzheimer, infection)
Liu et al., (2013) [14]<br>Vieira Lobato et al., (2015) [15]<br>Nehmi et al., (2021) [16]	Studies with streptozotocin-induced-diabetes
Saikia et al., (2018) [17]<br>Guerrero-Bonmatty et al, (2020) [18]<br>Yang et al., (2021) [19]	Mixtures
Everard et al., (2014) [20]<br>Albuquerque et al., (2019) [21]	Probiotics
Anugraheni et al., (2020) [22]	Body weight not evaluated
Erisgin et al., (2018) [23]	Short BG exposition

References:

[1] Kuroiwa Y, Nishikawa A, Imazawa T, Kanki K, Kitamura Y, Umemura T, et al. Lack of subchronic toxicity of an aqueous extract of Agaricus blazei Murrill in F344 rats. Food Chem Toxicol 2005;43:1047–53. https://doi.org/https://doi.org/10.1016/j.fct.2005.02.007.

[2] Kimura Y, Sumiyoshi M, Suzuki TT, Suzuki TT, Sakanaka M. Inhibitory effects of water-soluble low-molecular-

weight β-(1,3–1,6) d-glucan purified from Aureobasidium pullulans GM-NH-1A1 strain on food allergic reactions in mice. Int Immunopharmacol 2007;7:963–72. https://doi.org/https://doi.org/10.1016/j.intimp.2007.03.003.

[3] Chen SN, Nan FH, Chen S, Wu JF, Lu CL, Soni MG. Safety assessment of mushroom β-glucan: subchronic toxicity in rodents and mutagenicity studies. Food Chem Toxicol 2011;49:2890–8. https://doi.org/10.1016/j.fct.2011.08.007.

[4] Ubaidillah NHN, Abdullah N, Sabaratnam V, Hafizah N, Ubaidillah NHN, Abdullah N, et al. Isolation of the intracellular and extracellular polysaccharides of Ganoderma neojaponicum (Imazeki) and characterization of their immunomodulatory properties. Electron J Biotechnol 2015;18:188–95. https://doi.org/https://doi.org/10.1016/j.ejbt.2015.03.006.

[5] Chen SN, Chang CS, Chen S, Soni M. Subchronic toxicity and genotoxicity studies of Antrodia mushroom β-glucan preparation. Regul Toxicol Pharmacol 2018;92:429–38. https://doi.org/https://doi.org/10.1016/j.yrtph.2017.12.022.

[6] Silva AZ, Costa FPL, Souza IL, Ribeiro MC, Giordani MA, Queiroz DA, et al. Botryosphaeran reduces obesity, hepatic steatosis, dyslipidaemia, insulin resistance and glucose intolerance in diet-induced obese rats. Life Sci 2018;211:147–56. https://doi.org/https://doi.org/10.1016/j.lfs.2018.09.027.

[7] Shin HD, Yang KJ, Park BR, Son CW, Jang HJ, Ku SK. Antiosteoporotic effect of Polycan, β-glucan from Aureobasidium, in ovariectomized osteoporotic mice. Nutrition 2007;23:853–60. https://doi.org/10.1016/j.nut.2007.08.011.

[8] Miranda CCBO, Dekker RFH, Serpeloni JM, Fonseca EAI, Cólus IMS, Barbosa AM. Anticlastogenic activity exhibited by botryosphaeran, a new exopolysaccharide produced by Botryosphaeria rhodina MAMB-05. Int J Biol Macromol 2008;42:172–7. https://doi.org/https://doi.org/10.1016/j.ijbiomac.2007.10.010.

[9] Shi S-HH, Yang W-TT, Huang K-YY, Jiang Y-LL, Yang G-LL, Wang C-FF, et al. β-glucans from Coriolus versicolor protect mice against S. typhimurium challenge by activation of macrophages. Int J Biol Macromol 2016;86:352–61. https://doi.org/10.1016/j.ijbiomac.2016.01.058.

[10] Shao S, Wang D, Zheng W, Li X, Zhang H, Zhao D, et al. A unique polysaccharide from Hericium erinaceus mycelium ameliorates acetic acid-induced ulcerative colitis rats by modulating the composition of the gut microbiota, short chain fatty acids levels and GPR41/43 respectors. Int Immunopharmacol 2019;71:411–22. https://doi.org/10.1016/j.intimp.2019.02.038.

[11] Gudi R, Suber J, Brown R, Johnson BM, Vasu C. Pretreatment with Yeast-Derived Complex Dietary Polysaccharides Suppresses Gut Inflammation, Alters the Microbiota Composition, and Increases Immune Regulatory Short-Chain Fatty Acid Production in C57BL/6 Mice. J Nutr 2020;150:1291–302. https://doi.org/10.1093/jn/nxz328.

[12] Hiramoto K, Nishioka J, Suzuki K. Innate immune activation and antitumor effects of Lactobacillus-fermented Sparassis crispa extract in mice. J Funct Foods 2020;75:104215. https://doi.org/10.1016/j.jff.2020.104215.

[13] Xu M, Mo X, Huang H, Chen X, Liu H, Peng Z, et al. Yeast β-glucan alleviates cognitive deficit by regulating gut microbiota and metabolites in Aβ1–42-induced AD-like mice. Int J Biol Macromol 2020;161:258–70. https://doi.org/10.1016/j.ijbiomac.2020.05.180.

[14] Liu Y, Sun J, Rao S, Su Y, Li J, Li C, et al. Antidiabetic activity of mycelia selenium-polysaccharide from Catathelasma ventricosum in STZ-induced diabetic mice. Food Chem Toxicol 2013;62:285–91. https://doi.org/https://doi.org/10.1016/j.fct.2013.08.082.

[15] Vieira Lobato R, De Oliveira Silva V, Francelino Andrade E, Ribeiro Orlando D, Gilberto Zangeronimo M, Vicente de Sousa R, et al. Metabolic effects of β-glucans (Saccharomyces cerevisae) per os administration in rats with streptozotocin-induced diabetes. Nutr Hosp 2015;32:256–64. https://doi.org/10.3305/nh.2015.32.1.9013.

[16] Nehmi VA, Murata GM, Moraes RCM de, Lima GCA, De Miranda DA, Radloff K, et al. A novel supplement with yeast β-glucan, prebiotic, minerals and Silybum marianum synergistically modulates metabolic and inflammatory pathways and improves steatosis in obese mice. J Integr Med 2021;19:439–50. https://doi.org/10.1016/j.joim.2021.05.002.

[17] Saikia D, Manhar AK, Deka B, Roy R, Gupta K, Namsa ND, et al. Hypocholesterolemic activity of indigenous probiotic isolate Saccharomyces cerevisiae ARDMC1 in a rat model. J Food Drug Anal 2018;26:154–62. https://doi.org/https://doi.org/10.1016/j.jfda.2016.12.017.

[18] Guerrero-Bonmatty R, Gil-Fernández G, Rodríguez-Velasco FJ, Espadaler-Mazo J. A Combination of Lactoplantibacillus plantarum Strains CECT7527, CECT7528, and CECT7529 Plus Monacolin K Reduces Blood Cholesterol: Results from a Randomized, Double-Blind, Placebo-Controlled Study. Nutrients 2021;13:1206. https://doi.org/10.3390/nu13041206.

[19] Yang Y, Ye H, Zhao C, Ren L, Wang C, Georgiev MI, et al. Value added immunoregulatory polysaccharides of Hericium erinaceus and their effect on the gut microbiota. Carbohydr Polym 2021;262:117668. https://doi.org/10.1016/j.carbpol.2021.117668.

[20] Everard A, Matamoros S, Geurts L, Delzenne NM, Cani PD. Saccharomyces boulardii Administration Changes Gut Microbiota and Reduces Hepatic Steatosis, Low-Grade Inflammation, and Fat Mass in Obese and Type 2 Diabetic db/db Mice. MBio 2014;5. https://doi.org/10.1128/mBio.01011-14.

[21] Albuquerque RCMF, Brandão ABP, De Abreu ICME, Ferreira FG, Santos LB, Moreira LN, et al. Saccharomyces boulardii Tht 500101 changes gut microbiota and ameliorates hyperglycaemia, dyslipidaemia, and liver inflammation in streptozotocin-diabetic mice. Benef Microbes 2019;10:901–12. https://doi.org/10.3920/BM2019.0056.

[22] Anugraheni I, Andarini S, Handayani D, Wihastuti TA. Black yeast beta glucan for insulin resistance prevention through IL-33, ST2 and leptin Level: An In vivo study of an obesity model using Sprague dawley rats. Res J Pharm Technol 2020;13:6077–80. https://doi.org/10.5958/0974-360X.2020.01059.8.

[23] Erisgin Z, Takir S. The effect of beta glucan on MTX induced testicular damage in rats Investigation of the vascular effects of Alchemilla vulgaris liquid extracts View project 2018. https://doi.org/10.1080/10520295.2017.1391407.
